# Vulnerability to simple faints is predicted by regional differences in brain anatomy

**DOI:** 10.1016/j.neuroimage.2009.05.038

**Published:** 2009-09

**Authors:** Felix D.C.C. Beacher, Marcus A. Gray, Christopher J. Mathias, Hugo D. Critchley

**Affiliations:** a Clinical Imaging Sciences Centre, Brighton and Sussex Medical School (BSMS), Brighton, BN1 9RY, UK; b Division of Neurosciences and Mental Health, Imperial College London, London SW7 2AZ, UK

**Keywords:** Neurocardiogenic syncope, Vasovagal syncope, Blood pressure, Brain anatomy, Magnetic Resonance Imaging, Voxel-based morphometry

## Abstract

Neurocardiogenic syncope (NCS, simple fainting) is a common and typically benign familial condition, which rarely may result in traumatic injury or hypoxic convulsions. NCS is associated with emotional triggers, anxiety states and stress. However, the etiology of NCS, as a psychophysiological process, is poorly understood. We therefore investigated the relationship between NCS and brain anatomy. We studied a non-clinical sample of eighteen individuals with histories characteristic of NCS, and nineteen matched controls who had never fainted. We recorded fainting frequency, resting heart rate variability measures and anxiety levels. Structural T1-weighted magnetic resonance images (MRI) were acquired at 1.5 T. Associations between brain morphometry (regional gray and white matter volumes) and NCS, resting physiology and anxiety were tested using voxel-based morphometry (VBM). Compared to controls, NCS participants had lower regional brain volume within medulla and midbrain (*a priori* regions of interest). Moreover, across NCS individuals, lower gray matter volume in contiguous regions of left caudate nucleus predicted enhanced parasympathetic cardiac tone, fainting frequency and anxiety levels. Our findings provide preliminary evidence for a hierarchical anatomical basis to NCS. First, differences in the volume of brainstem centers supporting cardiovascular homeostasis may relate to constitutional predisposition to NCS. Second, differences in the structural organization of the caudate nucleus in NCS individuals may relate to fainting frequency via interactions between emotional state and parasympathetic control of the heart. These observations highlight the application of VBM to the identification of neurovisceral mechanisms relevant to psychosomatic medicine and the neuroscience of emotion.

## Introduction

Neurocardiogenic syncope (NCS; simple fainting or ‘vasovagal’ syncope) is a syndrome in which a neural reflex (involving an increase in vagal activity and an associated reduction of sympathetic drive) leads to a critical decrease of cerebral blood flow, resulting in a temporary loss of consciousness. Syncope is a common problem, affecting up to approximately 35% of the general population at least once (Serletis et al., 2006) and NCS is the most common fainting condition, accounting for 30–40% of all syncope cases (Sarasin et al., 2001). NCS is frequently familial (Mathias et al., 1998) however the genetics of NCS are largely unknown. NCS is typically first manifested in adolescence, with the reported median onset age of 14 years. Among people who express NCS, approximately 83% have fainted at least once by the age of 20 years (Serletis et al., 2006). NCS is distinguishable from other forms of syncope by this relatively early onset, by a higher proportion of females, a generally benign course and, most characteristically, by its association with specific ‘emotional’ triggers that include social challenge in people with social anxiety and the sight of blood or penetrative injury in people with blood phobia (Mathias et al., 2001).

Faints are not only triggered by these salient emotional challenges but are also influenced by affective factors. People who experience recurrent NCS have higher rates of anxiety (30%), panic (20%) and depression (15%), compared to the general (non-syncopal) population (Kouakam et al., 2002; Cohen et al., 2000). However, the causal relationship between cognitive/affective style and NCS is unclear and there may exist a bidirectional relationship between cognitive/affective factors and syncopal symptoms in people with NCS (Gracie et al., 2006).

Diagnosis of NCS is based primarily on a detailed history which examines personal and family record of cardiovascular or neurological disease, in order to exclude these as a possible cause. Also relevant are the frequency and circumstances of faints including other triggers; NCS is not associated with defecation, urination, coughing, swallowing or other neck movements. Where there is diagnostic uncertainly, assessment of these atypical cases may require further investigation, for example tilt table testing (Grubb, 2005). Individuals who experience NCS may have recurrent faints and more commonly experience transient presyncopal symptoms including dizziness and nausea. Syncope in response to emotional triggers is also more likely to occur in certain situations, including hot environments and after prolonged standing (orthostatic stress; Brignole et al., 2004). While NCS is usually benign, it may nevertheless impact significantly on quality of life and cause traumatic injury (Silverstein et al., 1982). Also, repeated faints may indicate the presence of a more substantive medical disorder and consequently investigational costs of fainting are high (Mathias et al., 2001).

Syncope is ultimately caused by a sudden reduction in cerebral blood flow to less than approximately half of its normal value (Kapoor, 2000). In the periphery, there is a biphasic cardiovascular response, with first a vasopressor phase, involving increased blood pressure and increased heart rate, followed by a vasodepressor phase, involving a withdrawal of sympathetic vasoconstrictor outflow and increased parasympathetic tone (Jardine et al., 1998) producing peripheral vasodilation and bradycardia. A decrease in vascular resistance causes a drop in blood pressure such that cerebral perfusion is compromised. This is usually the main mechanism of syncope (Hainsworth, 2004). Nevertheless, there is individual variability, leading to ‘vasodepressor’, ‘cardioinhibitory’ or ‘mixed’ subcategories of neurocardiogenic faints (Steptoe and Wardle, 1988; Ost et al., 1984; Sutton et al., 1992). A key component to the short-term regulation of systemic blood pressure is the baroreflex: Low systemic blood pressure activates aortic and carotid baroreceptors that trigger reflex adjustments to efferent sympathetic and parasympathetic outflows to raise blood pressure through increased vasoconstriction and cardiac output. The baroreflex is mediated via brainstem centers including the nucleus of the solitary tract (NTS), caudal ventrolateral medulla (CVLM) and rostral ventrolateral medulla (RVLM).

Sharpey-Schafer (1956) proposed a ‘ventricular theory’ of NCS which posits that an (emotion induced) attempt to increase cardiac output under conditions of low venous return/ventricular filling results in pronounced pressure transients in the myocardial wall. These pressure transients are relayed to the brainstem via vagus nerve afferents and lead to a burst of parasympathetic vagal activity, causing rapid bradycardia and peripheral vasodilation, thereby critically lowering systemic blood pressure. There is some empirical support for this theory (Mark, 1983; Oberg and Thoren, 1972; Lee et al., 1996; Shalev et al., 1991). However, other factors may also contribute significantly. Notably, people with NCS demonstrate abnormalities in cerebral autoregulation, where active cerebral vasoconstriction occurs in response to low systemic blood pressure (e.g. Grubb et al., 1991), in contrast to a normal adaptive *reduction* of cerebrovascular resistance. Heightened sympathetic reactivity also may contribute to the expression of NCS: People with blood phobia-related NCS show exaggerated baroreceptor-related inhibition of muscle sympathetic nerve activity (MNSA) controlling vasoconstriction within muscle vascular beds muscle in response to strong electrocutaneous stimulation (Donadio et al., 2007) and may show abnormally low MSNA during orthostatic challenge (Morillo et al., 1997). Others point to abnormalities in baroreceptor sensitivity (Ellenbogen et al., 1997; Mosqueda-Garcia et al., 1997) and neurohumoral factors (Robertson et al., 1999) in NCS. In summary, while the pathoetiology of NCS remains uncertain, current data suggest abnormal interaction between neural systems linked to emotional processing and brainstem mechanisms mediating baroreflexive control of blood pressure.

In order to elucidate further the neurobiological basis to this common psychophysiological condition, we examined the relationship between regional brain volume and fainting frequency, peripheral physiology and anxiety levels in people with a history of NCS and controls. Specifically, we hypothesised on the basis of prior studies of patients with acquired syncopal conditions (e.g. Minnerop et al., 2007) that NCS would be associated with volume decreases in brainstem regions mediating blood pressure regulation (and, by extension, that these brainstem volume reductions would be significantly negatively correlated with syncopal frequency). We further hypothesised that other differences in regional brain volume (increases or decreases) would reflect the interaction of physiological and affective vulnerabilities to syncope, with a more direct relationship to syncopal frequency. We used voxel-based morphometry (VBM) for unbiased (user independent) computerised comparison of regional brain anatomy in people with a history of NCS and controls.

## Materials and methods

### Participants

We studied a non-clinical sample of eighteen individuals with a history of NCS (mean age 28.6 years; 14 F, 4 M; 15 right handed) and nineteen (age and gender) matched controls (mean age 27.6 years; 14 F, 5 M; 18 right handed; see Table 1). The two groups were also matched on levels of anxiety and depressive mood symptoms. Syncope was defined as a transient loss of consciousness with spontaneous recovery. NCS was attributed as the basis of faints after detailed participant interview, confirming onset in childhood or early adulthood, family history and the presence of characteristic triggers e.g. emotional stress or exposure to blood stimuli. Two individuals reported faints only in the context of orthostatic stress, and four individuals only in the context of mild illness. Two had no clear trigger to single faints. Based on published data (Serletis et al., 2006) we calculated the probability of each control participant going on to express NCS in the future (i.e. that controls were misclassified). The mean risk of misclassification was 7.4% (range: 1% to 12.5%). In all individuals, and particularly those with no identifiable trigger, NCS was inferred if there was no suggestion of cardiological or neurological disease, and if syncopal episodes occurred in the absence of defecation, urination, coughing, or swallowing. We excluded people with previous history of neurological dysfunction and physical or psychiatric disorder affecting brain function, or known history of head injury. We also excluded participants who were taking psychotropic medication at the time of the study. We excluded one person from the NCS group from the analysis, on the basis of gross abnormality in brain anatomy. All participants underwent a structured health interview, and completed a self-report scale (Body Perception Questionnaire; Porges, 1993), which includes a medical history. Four individuals in the NCS group and one in the control group had a family history (first degree relative) of NCS. The project was approved by the Local Ethics Committee, and after complete description of the study to the participants written informed consent was obtained from all participants.

### Physiological measurements

We screened all participants using laboratory measures of resting supine heart rate and derived heart rate variability indices of sympathetic and parasympathetic activities. This further ensured that we could exclude participants with autonomic failure, cardiac dysfunction or rarer non-NCS conditions associated with syncope (see Brignole et al., 2004). Heart rate data was acquired immediately before structural scanning with participants still and reclined in the scanner for 5 min using a pulse oximeter (Nonin 8600FO). We measured resting mean and standard deviation of interbeat interval (IBI) and relative percentages of high frequency (0.14–4 Hz; HF-HRV) and low frequency (0.03–0.14 Hz; LF-HRV) heart rate variability (HRV analysis tool version 2.0 http://bsamig.uku.fi/index.shtml, Department of Physics, University of Kuopio, Finland). No outliers were found in either the NCS or control groups on these measures (in NCS, this could have indicated cardiac syncope).

### Questionnaire measurements

Alongside the Body Perception Questionnaire, which was used for an adjunct to health screening, we quantified subjective anxiety levels using the Beck Anxiety Inventory (BAI; The Psychological Corporation) and levels of depression using the Beck Depression Inventory (BDI; The Psychological Corporation). For each participant the BAI and BDI were completed on the day of scanning.

### MRI protocol

Participants underwent structural MRI imaging using a Siemens Avanto 1.5 T MRI scanner (Siemens, Erlangen, Germany) at the Clinical Imaging Sciences Centre (CISC), Brighton and Sussex Medical School. A vacuum fixation device ensured that participants were both comfortable and restrained from movement during the scanning process.

Whole brain volumes were acquired using a sagittal three-dimensional T1-weighted magnetisation-prepared rapid acquisition gradient echo sequence (MPRAGE, 192 slices, 11.4/4.4 ms TR/TE, 300 ms inversion time, 250 × 250 mm^2^ FOV, 0.9 mm isometric voxels). Acquisition time was 4 min, 58 s.

### Voxel Based Morphology (VBM) methodology

VBM permits the automated user-independent voxel-wise measurement of associations between regional brain volumes across groups of individuals and individual differences on other measures, including disease status. In this context, VBM has been extensively cross-validated with manual volumetric analysis (e.g. Woermann et al., 1999). The technique derives volume measurements from transformation of individual structural MR volume images into a common stereotactic space permitting testing of differences in sub-volumes of distinct brain regions using general linear model statistics. Our VBM methodology capitalized on developments of earlier methodologies, notably optimized VBM (Good et al., 2001). Volume brain images were first manually adjusted to a common orientation with the origin at the anterior commissure before all images were pre-processed using established VBM methods and SPM8b software (Wellcome Trust Centre for Neuroimaging, Institute of Neurology, UCL, London UK, http://www.fil.ion.ucl.ac.uk/spm; Ashburner and Friston, 2000), on a MATLAB 6.5.1 platform (The MathWorks, MA, USA): Three-dimensional T1-weighted MPRAGE images were visually examined for artefacts and structural abnormalities. Images were then segmented into gray matter, white matter and cerebrospinal fluid, and again visually inspected. Gray and white matter images were normalized to gray matter templates generated from all participants using a diffeomorphic registration algorithm (DARTEL; Ashburner, 2007). This non-linear warping technique minimises structural variation between participants and improves the sensitivity of VBM analysis, particularly for small inner brain structures (Bergouignan et al., 2009; Yassa and Stark 2009). A modulation step was used to permit voxel-wise information about local tissue volume (Ashburner and Friston, 2000). Tissue probability maps were obtained by averaging across participant data, using an 8-mm FWHM Gaussian smoothing kernel. Measures of total intracranial volume were obtained from summed global signal of segmented images of gray and white matter and cerebrospinal fluid.

The results of the segmentation process were visually examined for accuracy. This examination showed that the medulla was segmented predominantly as gray matter, and the pons and the midbrain as white matter. Accordingly, we used separate masks for brainstem regions-of-interest. These consisted of; 1. medulla (consisting of a vertically aligned 10 × 10 × 15 mm cuboid (volume = 1500 mm^3^), centered at (0 − 39 − 59), designed to ensure full coverage of the medulla); 2. pons (volume = 2157 mm^3^; using a template from the WFU PickAtlas; Maldjian et al., 2003), and; 3. midbrain (volume = 2289 mm^3^; also based on the WFU PickAtlas template). Anatomical localisation of the cerebral areas expressing differences in tissue volume was performed using a 3D anatomical atlas for SPM (Eickhoff et al., 2005).

### Statistical analyses

Two-tailed Pearson correlations were employed in analyses of physiological and questionnaire data. Voxel-wise comparisons of spatially normalized gray and white matter maps were made using SPM8b and considered separately. Group comparisons were performed using an analysis of covariance (ANCOVA) model, with total intracranial volume and age included as confounding covariates. For *a priori* regions-of-interest (ROIs), we used masks in order to permit less stringent correction for multiple comparisons.

We required significant volume differences to survive correction for multiple comparisons for the volume (*p* < 0.05 using cluster level significance).

Despite having directional hypotheses for differences in our ROIs, for rigor, we used two-tailed tests of significance for our ROIs. Threshold significance for regional differences in brain volume outside of *a priori* ROIs was set at *p* < 0.001 (uncorrected, but using two-tailed *t* tests to test associations in either direction). Also, we only considered clusters of 10 voxels or greater.

First, we tested for regionally specific group differences in gray and white matter between NCS and control participants using two-tailed *t* tests. Second, we tested across the NCS group for relationships between whole brain gray matter volume and fainting frequency. Third, following the observation that across NCS individuals fainting frequency was related (linearly and non-linearly) to parasympathetic power in heart rate variability, we explored relationships between whole brain gray matter volume and physiological measures (high frequency heart rate variability), within and between NCS and control groups. Last, we tested for relationships between regional brain volume and reported anxiety levels within and between the NCS and control groups.

## Results

### Fainting frequency

Participants were included in the NCS group if they had experienced one or more faints over the course of their lifetimes, where description of faint was consistent with NCS and there was no other plausible account for the syncope. Within the NCS group, the number of fainting incidents per year (fainting frequency) ranged from 0.023 to 0.522, with a mean of 0.120 and standard deviation of 0.13. Fainting frequency significantly deviated from a normal distribution. Logarithmic transformation of fainting frequency (log[fainting frequency]) produced a normal distribution with no outliers.

### Physiological and questionnaire data

Consistent with the matching of NCS and control groups, there was no significant group difference in reported anxiety or depression levels (mean ± SD: BAI score: NCS group 11.7 ± 10, controls 8.2 ± 6; BDI score: NCS group 3.6 ± 3, controls 5 ± 7). No significant relationship was observed between fainting frequency or log[fainting frequency] and depressive symptoms. Across all participants (*N* = 37; assigning controls a score of 0 faints), fainting frequency was significantly positively correlated with anxiety level [*r* = 0.36, *p* = 0.03]. Within the NCS group (*n* = 18) similar correlation coefficient magnitudes were observed for correlations between BAI and both fainting frequency [*r* = 0.34] and log[fainting frequency] [*r* = 0.35], although these correlations did not reach criterion significance.

Across all participants there was no significant correlation between anxiety levels and HF-HRV. We also found no significant group differences in resting heart rate (mean ± SD of resting IBI: NCS group 848 ms ± 133 ms, control group 832 ms ± 125 ms). Derived heart rate variability measures were obtained to index relative sympathetic (LF-HRV; 0.04–0.15 Hz) and parasympathetic (HF-HRV; 0.15–0.4 Hz) powers. These measures were assessed via an autoregressive (AR) parametric model for spectrum estimation (HRV analysis tool version 2.0). No group differences were observed at rest in sympathetic power (% mean ± SD of LF-HRV: NCS group 43.6 ± 16, control group 43.2 ± 19) or parasympathetic power (vagus nerve) (% mean ± SD of HF-HRV: NCS group 53.9 ± 17, control group 54.6 ± 19). There were no outliers in the HRV data. Together these results are consistent with reported evidence suggesting that there are no characteristic differences between NCS and control groups in cardiac autonomic measures at rest (Piccirillo et al., 2004).

We next explored whether relationships existed between autonomic physiological parameters and the occurrence of fainting in people vulnerable to NCS. Within the NCS group, LF-HRV was significantly negatively correlated with fainting frequency [*r* = − 0.56, *p* = 0.018] and log[fainting frequency] [*F* = 12.64, *p* = 0.003, *R*^2^ = 0.46]. Within the NCS group, HF-HRV was significantly positively correlated with fainting frequency [*r* = − 0.54, *p* = 0.026] and log[fainting frequency] [*F* = 10.96, *p* = 0.005, *R*^2^ = 0.42] Fig. 2B.

### Brain morphometry and NCS

#### Group differences

**Fig. 1:**
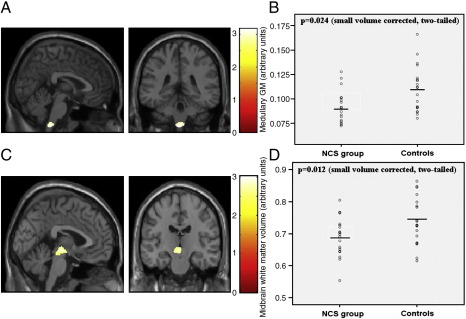
(A) Significant differences (two-tailed) in medullary gray matter volumes in individuals with a history of NCS (*n* = 18) and controls (*n* = 19), superimposed on T1-weighted images (medullary region centered at − 5, − 39, − 62). (B) Scatter plot of medullary gray matter for NCS and control groups, showing group means. (C) Significant differences (two-tailed) in midbrain white matter volumes in NCS individuals and controls. (D) Scatter plot of medullary gray matter for NCS and control groups, showing group means.

There were significant group differences in regional gray matter volume (Table 2). Notably, NCS participants had significantly lower volume within a midline medullary cluster compared to controls (small volume corrected t test [*t*(1,33) = 3.14, *p* = 0.024, two-tailed]; whole brain uncorrected significance was [*t*(1,33) = 3.14, *p* = 0.002, two-tailed]; Fig. 1).

There were significant group differences in regional white matter volume (Table 2).

NCS participants had significantly lower volume within a midbrain cluster compared to controls (small volume corrected *t* test [*t*(1,33) = 2.82, *p* = 0.012, two-tailed]; whole brain uncorrected significance was [*t*(1,33) = 2.82, *p* = 0.002, two-tailed]; Fig. 1).

No other brain region showed significant decreases in volume in the NCS group. Additional brain regions showed group differences, in gray matter volume, in the opposite direction. Greater regional gray matter volumes were observed in NCS compared to controls in bilateral inferior temporal and supramarginal gyri. Greater white matter volume was also observed in occipital and inferior parietal regions of NCS individuals (Table 2).

#### Fainting frequency, physiology and regional gray matter volume

**Fig. 2:**
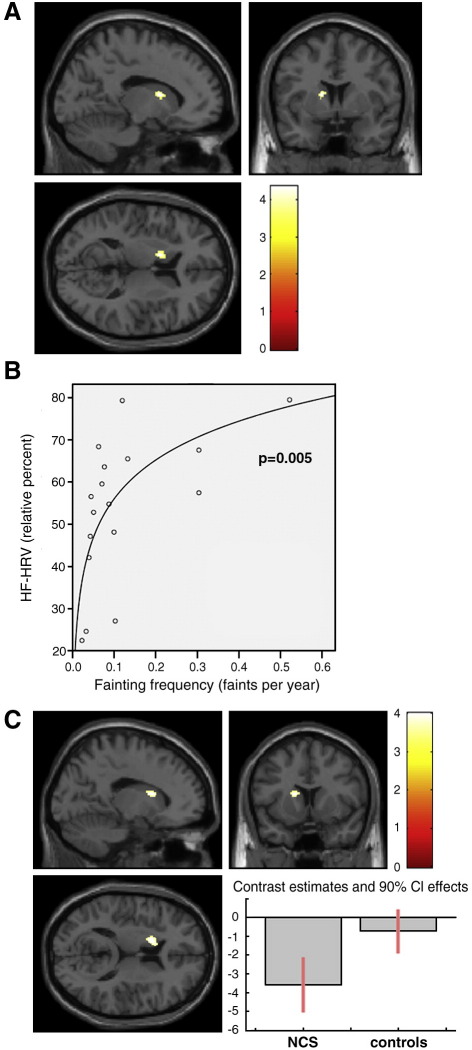
(A) Left caudate region showing significant negative correlations between regional gray matter volumes and fainting frequency, within NCS participants. (B) Significant negative logarithmic relationship between HF-HRV and fainting frequency, within NCS participants [*p* = 0.005, *R*^2^ = 0.422]. (C) Left caudate region showing significant negative correlations between regional gray matter volumes and HF-HRV, within NCS participants. Contrast estimates reveal this correlation was driven primarily by NCS participants.

In a further analysis, we directly explored the relationship between regional gray matter volume and fainting frequency, within NCS individuals using voxel-wise regression analyses. We observed a significant negative relationship between left caudate gray matter volume and fainting frequency within the NCS group (Fig. 2A; Table 3). No other regions showed a significant negative relationship between volume and fainting frequency.

We also observed that volume of some brain regions were positively correlated with fainting frequency in NCS patients. These regions were secondary somatosensory cortex, dorsolateral prefrontal and caudal orbitofrontal cortices (Table 3).

We next explored the relationship between peripheral physiological response and regional gray matter volume, following the observation above that there were significant relationships between fainting frequency, log[fainting frequency] and heart rate variability measures of vagal parasympathetic tone within the NCS group (Fig. 2B). We regressed regional brain volume across participants against measures of resting heart rate variability. Because of the close coupling of relative HF-HRV and LF-HRV measures [*r* = − 0.98, *p* < 0.0001] we selected HF-HRV as the regressor of interest. Using this approach to examine gray matter correlates of cardiovascular control within the NCS group, only one region attained threshold significance, and this relationship was located in left caudate nucleus (Table 3 and Fig. 2C). Here reduced volume of caudate nucleus was associated with higher resting HF-HRV. Parameter estimates showed this effect within caudate was significant for NCS individuals [*t* = 4.10, *p* < 0.001], but not controls [*t* = 0.95, *p* = 0.825]. No brain region showed the opposite relationship with HF-HRV (i.e. greater volume predicting greater parasympathetic tone) within the NCS group.

**Fig. 3:**
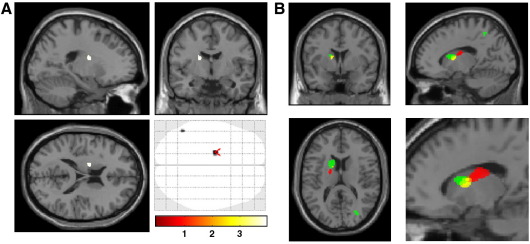
(A) caudate regions showing significant negative correlations between regional gray matter volumes and anxiety levels, within NCS participants. (B) Within NCS participants, left caudate regions showing significant negative correlations between regional gray matter volumes and anxiety levels (red), fainting frequency (yellow) and HF-HRV (green). These regions are also shown magnified and presented at *p* = 0.005.

Lastly, we tested relationships between gray matter volume and anxiety levels within and between NCS and control groups. Within both the NCS and control groups anxiety scores were found to significantly negatively correlate with gray matter volume within left caudate nucleus (Fig. 3A). Within the NCS group there were significant positive correlations between anxiety levels and gray matter volume of the right superior occipital gyrus and right superior orbital gyrus (Table 3).

In the light of our findings implicating regions of left caudate nucleus in fainting frequency, cardiac autonomic tone and anxiety level we plotted the anatomical relationship of these effects on a single template brain at a threshold of *p* < 0.005 (uncorrected) in order to illustrate the topographical contiguity of these effects (Fig. 3B4).

Within the NCS group there were no significant differences between individuals with and without a family history of NCS for volume of whole brain, total gray matter, total white matter, medulla or midbrain.

## Discussion

We found that people with a history of NCS manifest significant differences in regional brain volume within medulla and midbrain, compared to matched, non-fainting controls. Further, we found that subregional volumes within the left caudate nucleus were significantly negatively correlated with fainting frequency and resting heart rate variability measures in people with NCS, and with anxiety levels (in both NCS and controls). These observations are noteworthy since we also demonstrate an association between high frequency or “vagal” regulation of the heart (HF-HRV) and frequency of syncope in our NCS participants. Moreover, emotional state, notably anxiety, is well recognised as a predisposing factor to syncope in vulnerable NCS individuals (we observed an association with the occurrence of fainting across all our participants as a whole) and influences cardiovascular autonomic regulation (Friedman et al., 1993). Therefore one interpretation of our findings is that predisposition to NCS relates to differences in functional neuroarchitecture (expressed as differences in structural morphology) of brainstem regions mediating cardiovascular homeostasis, including baroreflex regulation of blood pressure. Further, our findings suggest that the likelihood of recurrent fainting is influenced at the level of left caudate nucleus, regions of which we found to be more strongly coupled to autonomic cardiac regulation in individuals with NCS than in controls, and which interact with adjacent regions, also within the caudate, which contribute to the expression of anxiety. These findings are novel and provide provisional insight into the mechanisms underlying NCS.

VBM techniques have historically lacked sensitivity to differences in brainstem regions. Nevertheless, significant findings within medulla, pons and midbrain are reported (often with small volume ROI approaches) both in patient groups and healthy populations (e.g. Uchida et al., 2008; Hauser et al., 2006; Ginestroni et al., 2008; Della Nave et al., 2008). Gray/white matter contrast within brainstem is typically lower on structural (T1-weighted) MR images than in supratentorial brain regions, due to the close proximity of fibre tracts and relatively small nuclei. Other factors such as brainstem movement also contribute to lower signal quality. In the present study the DARTEL VBM methodology enhanced our ability to detect brainstem differences. As with earlier instantiations of VBM, we found pons and midbrain regions to be predominantly segmented into white matter compartments and medulla into primarily gray matter compartments. Accordingly, we used separate masks for these brainstem ROIs.

NCS may reflect an adaptive survival mechanism in mammals that evolved to counter the effects of hemorrhage, but to which humans are particularly vulnerable while standing upright. In humans, brainstem regions are critical for autonomic regulation of blood pressure and cerebral blood flow (Verner et al., 2008; Henderson et al., 2000; Golanov and Reis, 2001). In the present study, the differences we observed between NCS and control groups in medullary volume encompassed two regions of particular interest: The nucleus of the solitary tract (NTS) which runs along the dorsal length of the medulla, and the caudal midline medulla (CMM). Pathological disturbance of the structural integrity of these nuclei could potentially affect a range of autonomic functions (in addition to blood pressure regulation) but importantly, no additional autonomic problems were present within our study group (healthy NCS individuals). Animal studies suggest potential mechanisms through which brainstem regions may contribute to syncope. The NTS is implicated in the control of both systemic and cerebral blood pressure, which decreases following excitatory amino acid injection into the NTS of anaesthetised rats (and this effect is abolished by local NMDA receptor blockade; Morino et al., 1994). The NTS can affect cerebral blood pressure via a ‘cerebral vasodilator area’ in the ventrolateral medulla (Golanov and Reis, 2001). Finally, the NTS also has excitatory efferent connections to brainstem nuclei of ascending monoamine neuromodulator pathways mediating central arousal and motivational attention. In close proximity to the NTS is the CMM which, like the NTS, receives visceral afferents including information from cardiovascular and arterial baroreceptors (e.g. Brodal 2004). A vasodepressor region is identified within the CMM where activation evokes bradycardia and hypotension (Verner et al., 2008), and where inactivation attenuates bradycardia and hypotension following hemorrhage (Henderson et al., 2000). Distinct regions within the CMM are differentially involved in the compensatory baroreflexive responses to hemorrhage (Heslop et al., 2002; Schadt and Ludbrook, 1991). Animal studies also implicate midbrain nuclei, including the ventrolateral column of the periaqueductal gray (vlPAG) in the response to hemorrhage, particularly in decompensatory vasodepressor reactions (Troy et al., 2003; Cavun and Millington, 2001). The vlPAG projects to medullary cardiovascular nuclei and critically drives the response of CMM during hemorrhagic shock (Vagg et al., 2008). Taken together, our brain morphometric data in humans link the neuroarchitecture of brainstem centers implicated in physiological reactions to low blood pressure and blood loss, to NCS, which is most classically expressed in the context of body penetration and blood phobias.

In addition to the specific nature of blood phobia, NCS is linked to more general emotional factors. We found that both fainting frequency and anxiety levels were significantly negatively correlated with volume of caudate nucleus. The caudate is central to the initiation and facilitation of voluntary control of movement, but is also functionally implicated in affective and motivational processes, including disgust reactions and anxiety disorders (Sprengelmeyer et al., 1995 Saxena et al., 2001, Swedo et al., 1992). In addition, several neuroimaging studies report decreases in caudate volumes associated with anxiety disorders, including obsessive compulsive disorder (e.g. Rubin et al., 1992; Robinson et al., 1995). Our results suggest that anxiety symptoms are related to differences in caudate gray matter and this in turn influences the expression of NCS. Although NCS is not associated with lower caudate volumes (we found no significant difference between NCS and control groups), our morphometric findings suggest that the caudate nucleus supports the modulatory influence of anxiety state on frequency of syncope in NCS, via a contribution to autonomic cardiovascular regulation. In both NCS and control participants, subregional volume within left caudate was associated with anxiety symptoms and this region abutted areas specifically linked to fainting frequency and cardiovascular parasympathetic tone in NCS individuals (but not controls). For this study, we intentionally matched the presence of anxiety symptoms across NCS and control groups. Nevertheless, this relationship between caudate volumes and the emotional component of NCS requires further investigation.

The association of left caudate volume with autonomic cardiac regulation within the NCS group (which interestingly was not present in controls), suggests that the occurrence of fainting may be influenced by differential central organization of cardiovascular control affecting physiological reactivity to emotional challenges. In our study, resting heart rate variability measures did not differ between the NCS and control groups, consistent with previous reports (Kochiadakis et al., 1997; Kochiadakis et al., 1998; Furlan et al., 1998; Morillo et al., 1994; Kouakam et al., 1999; Lipsitz et al., 1990; Piccirillo et al., 2004). Nevertheless, paced breathing can reveal increased vagal sinus modulation of heart rate (HF-HRV) which is not evident during free breathing (Piccirillo et al., 2004), suggesting that syncopal patients may have an increased sensitivity to vagal control. Importantly, however, we showed that the degree of parasympathetic modulation (percent relative HF-HRV power) of the heart at rest was positively associated (linearly and logarithmically) with fainting frequency within NCS individuals (and similarly that relative LF-HRV power was negatively associated with fainting frequency). Simply, individuals with increased parasympathetic modulation of the heart were more likely to have experienced neurocardiogenic faints, suggesting that people with NCS have an enhanced sensitivity to cardiovascular effects mediated via the vagus nerve. Most striking was the observation that the region of the left caudate whose volumes were associated with HF-HRV (in NCS patients but not controls) was adjacent to caudate regions for which volumes were associated with both fainting frequency and anxiety.

Within the NCS group other brain regions (including orbitofrontal cortex and motor cortices) showed independent associations with autonomic control of the heart, fainting frequency and anxiety levels. Within the NCS group we found a conjunction of associations between left caudate gray matter volume and three factors: Fainting frequency, anxiety level and HF-HRV. This conjunction provides compelling evidence that the neuroarchitecture of the neostriatum is an important contributing factor in the etiology and expression of NCS.

Functional brain mechanisms through which salient sensory challenges impact on blood pressure in non-fainting individuals engage the insula, amygdala, midbrain and brainstem regions, and are dependent on HF-HRV tone (Gray et al., 2009). Our VBM findings additionally implicate the caudate nucleus within NCS individuals. Further studies suggest that these reflex mechanisms underlying NCS may be distinct, depending on whether or not faints are phobia-related (Donadio et al., 2007). However, in the present study we did not have the statistical power to relate brain morphometry to specific syncopal triggers.

There are a number of methodological constraints on our study which require further comment. First, we took the straightforward approach of correlating brain morphometric measures with lifelong fainting frequency, in order to distinguish frequent fainters from a more usual pattern of only one or two syncopal episodes during the lifespan, typically in adolescence. We attempted to deal with age as a confounding factor within our analytic design, yet these approaches are problematic: After the study data were acquired, one participant, a medical student, reported three strong presyncopal events during a first attachment to a surgical/trauma service. Second, we used relatively young participants, increasing the possibility that our control group may have included individuals with a predisposition to NCS, but who have yet to experience a syncopal episode. However, this is unlikely to be a major confound for our analysis, as NCS is typically first manifested in early adolescence, with the reported median onset age of 14 years (Serletis et al., 2006). Based on published data (Serletis et al., 2006) we estimated that the mean risk of misclassification of our control participants was 7.4%. The selection of a relatively young sample has the advantage that it limits the extent to which age-related changes in brain pose a significant confound. Further, it limits the extent to which other acquired syncopal syndromes (such as autonomic failure and carotid sinus hypersensitivity) could confound our NCS group data. Our selection of a relatively young NCS sample was designed to balance these issues appropriately. Third, we studied a sample of individuals with subclinical NCS and with relatively low fainting frequency, potentially risking misclassification of participants into the NCS or control groups. However, we took steps in order to minimise this possibility, taking a comprehensive medical history and laboratory measurements of resting autonomic function. The possibility that some controls were misclassified involves risk of type 2 error, i.e. failure to detect real differences, or ‘false negatives’. However, this problem should not have compromised the reliability of the differences which we did detect, i.e. increased our vulnerability to type 1 error (‘false positives’). The use of a non-clinical NCS group minimises the risk that additional confounds related to clinical status, such as head injury or comorbid conditions, could influence our findings.

While our inferences regarding brain associations with syncope apply to the large section of the population who express neurocardiogenic faints, we do not expect that our results have implications for the diagnosis of NCS or for clinical management. No group differences in brain anatomy were apparent on visual inspection. Also, consistent with the overlap between groups in brainstem volumes (Figs. 1 and 2), the sensitivity and specificity for these measures, at an individual level, were low (for midbrain volume: 67% and 68% respectively).

We acknowledge that the statistical power of our study will likely improve with a larger sample size, and would, for example, permit an analysis of the influence of family history of NCS on brain morphometry. We nevertheless provide novel evidence for differences in brain morphometry in individuals with stereotyped patterns of autonomic reactivity associated with anxiety.

## Summary

Our study provides a first examination of the relationship between brain anatomy and symptomatology in NCS. Our results indicate that NCS, a stereotyped autonomic response tendency, is related to predisposing differences in brain anatomy in brainstem regions associated with blood pressure regulation. Moreover, our data indicate that within NCS individuals the architectural organization of subregions of caudate nucleus, related to autonomic cardiovascular regulation and experienced anxiety levels, influences the expression of faints. These findings illustrate the application of computerised analysis of brain morphometry in dissecting psychophysiological mechanisms underlying behavioural and emotional responsivity.

## Figures and Tables

**Table 1:** Demographic data, and syncope triggers of participants.

Demographics				Syncope trigger							
	Gender	Age	Family history of NCS	Orthostatic stress	Emotional stress	Blood related	Having minor medical procedure (e.g. injection)	Observing medical procedure	Pain	Tiredness/mild illness (e.g. travel sickness, a cold)	Unexplained
1	F	20	No							X	
2	F	22	No							X	
3	F	23	Yes	X				X		X	
4	F	23	No		X	X	X	X			X
5	F	23	No	X							
6	F	25	No							X	
7	F	25	No				X				
8	F	26	Yes						X		
9	F	28	No			X					
10	F	29	No								X
11	F	30	No			X				X	
12	F	30	No							X	
13	F	34	No				X	X			
14	F	39	No				X				X
15	M	23	Yes	X							
16	M	32	Yes						X		X
17	M	39	No								X
18	M	43	No	X		X					

**Table 2:** A summary of regions detected in the main effects analysis, with their MNI coordinates, extent thresholds and *t*-values.

Region	Side	Coordinates of cluster centroid	Cluster size	*t* value of peak
Gray matter: NCS > controls				
Infero-temporal cortex	L	− 62, − 28, − 27	1439	5.95
Infero-temporal cortex	R	63, − 31, − 24	574	4.29
Supramarginal gyrus	R	68, − 18, 24	172	4.70
White matter: NCS > controls				
Middle occipital lobe	L	− 32, − 85, 13	35	4.24
Inferior parietal lobe	L	− 32, − 51, 43	43	3.65

Differences are significant at a threshold of *p* < 0.0005 uncorrected (two-tailed, except for medullary ROI), for clusters of 10 or more contiguous voxels.

**Table 3:** Summary of correlations between gray matter volumes and heart rate variability fainting frequency, and anxiety levels, within NCS participants.

Region	Side	Coordinates of cluster centroid	Cluster size	*t* value of peak
NCS gray matter: negative correlations with fainting frequency				
Caudate nucleus	L	− 14, 8, 12	61	4.35
NCS gray matter: positive correlations with fainting frequency				
Somatosensory S2	R	55, − 19, 10	74	5.05
Dorsolateral prefrontal cortex	R	36, 11, 48	245	4.58
Parahippocampal gyrus	L	− 21, 9, 44	65	3.91
Orbitofrontal cortex	R	27, 27, − 15	104	4.21
NCS gray matter: negative correlations with anxiety score				
Caudate nucleus	L	− 20, − 6, 18	61	3.95
NCS gray matter: positive correlations with anxiety score				
Superior occipital gyrus	R	26, − 100, 10	11	3.73
Superior orbital gyrus	R	17, 48, − 18	110	3.67
NCS gray matter: positive correlations with LF-HRV				
Caudate nucleus	L	− 15, 9, 13	33	3.90
NCS gray matter: negative correlations with HF-HRV				
Caudate nucleus	L	− 15, 11, 13	76	4.10
NCS gray matter: negative correlations with HF-HRV				
Caudate nucleus	L	− 15, 11, 13	76	4.10

MNI coordinates, extent thresholds and peak *t*-values are included.
